# Standardised reports with a template format are superior to free text reports: the case for rectal cancer reporting in clinical practice

**DOI:** 10.1007/s00330-019-06028-8

**Published:** 2019-02-22

**Authors:** P. J. Brown, H. Rossington, J. Taylor, D. M. J. Lambregts, E. Morris, N. P. West, P. Quirke, D. Tolan, on behalf of the YCR BCIP Study Group

**Affiliations:** 1 Department of Clinical Radiology, Lincoln Wing, Leeds Teaching Hospitals NHS Trust, St James’ University Hospital, Beckett Street, Leeds, LS9 7TF UK; 2 Section of Epidemiology and Biostatistics, Leeds Institute of Cancer and Pathology, St James’s Institute of Oncology, St James’s University Hospital, University of Leeds, Leeds, LS9 7TF UK; 3 Department of Radiology, Netherlands Cancer Institute - Antoni van Leeuwenhoek, PO Box 90203, 1006 BE Amsterdam, Netherlands; 4 Section of Pathology and Tumour Biology, Leeds Institute of Cancer & Pathology, St James’s University Hospital, University of Leeds, Leeds, LS9 7TF UK

**Keywords:** Rectal cancer, Magnetic resonance imaging, Medical audit, Template-reporting

## Abstract

**Purpose:**

Rectal cancer staging with magnetic resonance imaging (MRI) allows accurate assessment and preoperative staging of rectal cancers. Therefore, complete MRI reports are vital to treatment planning. Significant variability may exist in their content and completeness. Template-style reporting can improve reporting standards, but its use is not widespread. Given the implications for treatment, we have evaluated current clinical practice amongst specialist gastrointestinal (GI) radiologists to measure the quality of rectal cancer staging MRI reports.

**Materials and methods:**

Sixteen United Kingdom (UK) colorectal cancer multi-disciplinary teams (CRC-MDTs) serving a population over 5 million were invited to submit up to 10 consecutive rectal cancer primary staging MRI reports from January 2016 for each radiologist participating in the CRC-MDT. Reports were compared to a reference standard based on recognised staging and prognostic factors influencing case management

**Results:**

Four hundred ten primary staging reports were submitted from 41 of 42 (97.6%) eligible radiologists. Three hundred sixty reports met the inclusion criteria, of these, 81 (22.5%) used a template. Template report usage significantly increased recording of key data points versus non-template reports for extra-mural venous invasion (EMVI) status (98.8% v 51.6%, *p* < 0.01) and circumferential resection margin (CRM) status (96.3% v 65.9%, *p* < 0.01). Local tumour stage (97.5% v 93.5%, NS) and nodal status (98.8% v 96.1%, NS) were reported and with similar frequency.

**Conclusion:**

Rectal cancer primary staging reports do not meet published standards. Template-style reports have significant increases in the inclusion of key tumour descriptors. This study provides further support for their use to improve reporting standards and outcomes in rectal cancer.

**Key Points:**

• *MRI primary staging of rectal cancer requires detailed tumour descriptions as these alter the neoadjuvant and surgical treatments.*

• *Currently, rectal cancer MRI reports in clinical practice do not provide sufficient detail on these tumour descriptors.*

• *The use of template-style reports for primary staging of rectal cancer significantly improves report quality compared to free-text reports.*

## Introduction

Magnetic resonance imaging (MRI) is the most accurate method of rectal cancer pre-operative staging and re-assessment [1–4]. Tumour features identified on the rectal cancer baseline staging MRI (‘primary staging’) determine the subsequent clinical management including whether neo-adjuvant radiotherapy or chemoradiotherapy (CRT) is given prior to surgical resection [1, 5]. Follow-up rectal cancer assessment MRI (‘restaging’) helps to determine the operative technique or alternative treatment approaches including the ‘watch and wait’ approach. The timing of imaging post neo-adjuvant CRT is debated, but is typically 6–8 weeks after completion of CRT [1, 5–9]. Imaging reports describe the tumour features to clinical teams influencing clinical decisions. This emphasises the importance of accurate and reproducible primary staging and restaging MRI reports.

There is increasing interest in structured reporting in radiology and pathology to improve communication of imaging findings and generating consistent reports, for clarity and content [10–13]. This applies to rectal MRI reporting with recent consensus statements published by the European Society of Gastrointestinal Abdominal Radiology (ESGAR) and Society of Abdominal Radiology (SAR) both recommending report templates for primary staging and restaging [14, 15]. Radiological imaging templates have been produced and evaluated elsewhere but often these templates have not been widely adopted, with many radiologists preferring free-text reports [16, 17]. Across 16 different hospital sites in UK (14 different NHS trusts) serving our population of 5.7 million, there is variable usage of template reporting in clinical practice for primary staging [18].

Across our population of approximately 1000 new rectal cancer diagnoses per year, we retrospectively evaluated the current standard of primary staging rectal cancer MRI reports in clinical practice [19].

## Materials and methods

This was a retrospective service evaluation study, so local ethical approval was waived. This study used only primary staging reports generated as a routine part of patients care. All reports were anonymised before entralisation to remove any patient identifiable information.

Sixteen UK colorectal cancer multi-disciplinary teams (CRC-MDTs) serving a combined population of over 5.7 million in Yorkshire, UK, were invited to participate. The CRC-MDT lead radiologist at each centre was invited by email to submit 10 consecutive primary staging reports for each radiologist in their department routinely reporting rectal MRI or regularly participating in the CRC-MDT. The anonymised consecutive reports had to be within a 12-month period from January 2016 until January 2017; therefore, those radiologists with a small workload of rectal cancer supplied all their primary staging reports for the year. All radiologists involved are gastrointestinal (GI) sub-specialists; all have received specialist training in rectal MRI and are members of either ESGAR or BSGAR.

Each site anonymised the clinical information, history and report content prior to submission. In addition, a coding system was employed by the lead radiologist at each site to allow unbiased analysis of each radiologist’s set of reports, whilst also allowing individualised feedback via the lead at each site.

Reports were compared by a single investigator to a reference standard based on key tumour descriptors from UICC-TNM 5 staging and other recognised factors known to influence case management that have subsequently been included in ESGAR and SAR recommendations [14, 15]. In total, the inclusion of 22 key tumour descriptors was evaluated within each report (Table 1). The inclusion of each tumour descriptor, or a comment confirming a negative finding within each report counted as ‘reported’. The failure to provide a description of the presence or absence of a feature in a report counted as ‘not reported’. Some appropriate report exclusions were allowed depending on the tumour features (e.g. the absence of a distance through the muscularis propria was considered acceptable for T1/T2 tumours or not stating which organs are involved by tumour with T1–3 staging). Several differing descriptive methods were permitted in reports for the relationship to the mesorectal fascia (MRF), and so depth of mesorectal fat invasion; either an absolute measurement or subcategories (i.e. T3a–d) and thus the potential risk to the circumferential resection margin (CRM) status.

**Table 1:** Tumour descriptors collected from each baseline rectal cancer staging MRI

Key tumour descriptor		Description of what the tumour descriptor assessed
Tumour	Vertical location	An indication of ‘lower’, ‘mid’ or ‘upper’ rectum
	Length	The vertical, unidirectional size of the tumour
	Distance from the anal verge	Measurement from anal verge to help plan the operation/radiotherapy
	Shape	A description of the tumour morphology, e.g. annular, semi-annular, polypoidal, flat
	Radial location of wall involvement	Inclusion of either a clock-face description or equivalent descriptive term (e.g. left lateral)
	MRI signal	A description of the predominant component (i.e. solid or mucinous tumour type)
	Relationship to peritoneal reflection	A description of the tumour relative to the peritoneal reflection; above, at the level of or below
	T stage	
If ≥ T3	Distance through muscularis propria/T3 subcategory	Inclusion of either a direct or indirect measurements; i.e. mm or T3 subcategories; T3^a–d^
MRF	MRF status	A description of if the MRF was threatened or involved
	If threatened/involved, by what	A description of what threatened/involved the MRF; i.e. tumour, EMVI, lymph node
	Minimum distance to the MRF	If threatened a measurement was required here, unless involved
	Location closest to MRF	Inclusion of descriptors of the location closest to the MRF; either a clock-face description or equivalent descriptive term (e.g. left lateral)
If ≥ T4	Involvement of peritoneum and/or which organs	A statement of which organs/ peritoneal involvement
Nodes	Nodal status	A statement of mesorectal or extra-mesorectal lymph node metastatic status
	If N+, location of involved nodes	A description of the involved lymph node location (i.e. mesorectal or extra-mesorectal)
	If N+, radial location of mesorectal nodal involvement	A description of the involved lymph node location (i.e. radial location for surgical planning)
	If N+, superior location of node involvement	A description of the involved lymph node location (i.e. radial location for surgical/radiotherapy planning)
EMVI	EMVI status	A statement of EMVI involvement (i.e. present or not)
	EMVI radial and/or superior location	A description of the involved lymph node location (e.g. radial location for surgical planning)
Metastases	Distant metastatic status	A statement on metastatic status if liver sequences included in the imaging protocol or known from other cross-sectional imaging assessment
Overall predicted TNM stage		

*MRF* mesorectal fascia, *EMVI* extra-mural venous invasion

To allow comparisons between reporting styles, the template or free-text style of reporting was also recorded.

A simple report scoring system for overall report quality was used based on the accumulated inclusion (or lack) of each key tumour descriptor giving a maximum score of 22. This was adjusted to calculate a ‘completeness’ percentage score for each reporter which corrected for case sets which appropriately excluded a tumour descriptor.

## Statistical analysis

All data was tabulated in Microsoft Excel (Office 365, Microsoft Corp.) and statistical analysis performed using Stata Statistical Software (Release 15, StataCorp LLC.). Fisher’s exact test was used to test for statistical significance in differences in reporting standards between free-text and template reports. Corrections for multiple testing were performed using Holm’s method of correction [20]; therefore, a corrected *p* value < 0.01 was required for consideration of statistical significance.

## Results

Four hundred ten primary staging reports were submitted from 41 of 42 (97.6%) eligible radiologists across the region. One trust (one radiologist) did not participate. Fifty reports (12.2%) were excluded; 16 reports as they were not pre-intervention MRI scans (of these, 14 were re-staging scans and 2 were baseline scans that acquired post-total excision biopsy), 4 were scans for non-rectal lower gastrointestinal tract tumours and 2 were rectal MRI for benign indications. A further 28 ‘potential reports’ were not analysed as they fell outside the 1 year data collection window. A total of 360 primary staging reports were included for analysis (median 10 per radiologist, Inter-Quartile Range 6–10, range 4–10 reports per radiologist); 81 (22.5%) were reported using intra-departmental standardised templates; this involved two different hospital organisations (a large teaching hospital and smaller district general hospital), the remaining 279 reports were free-text reports.

## Standard of report contents

There was substantial variability of tumour descriptor inclusion in reports (Table 2). Certain variables were reported in over 85% of all reports, including vertical location of tumours, tumour length, tumour and nodal staging and location of involved lymph nodes. However, other tumour descriptors were included in less than 75% of reports, including radial location of wall involvement by the tumour, distance through the muscularis propria (for tumours > T3; 229 reports were of T3 or T4 tumours), MRF status and extra-mural venous invasion status. The tumour descriptors with the lowest likelihood of being included in primary staging reports were the tumour relationship to the peritoneal reflection and location of the most superior malignant mesorectal lymph node (relative to the sacral level), which are important surgical and radiotherapy landmarks respectively.

**Table 2:** Tumour descriptors and their inclusion in the total number of reports

		Total number of reports including the variable/total number of reports (%)
Tumour	Vertical location	327/360 (91%)
	Length	312/360 (87%)
	Distance from the anal verge	305/360 (85%)
	Shape	260/360 (72%)
	Radial location of wall involvement*	156/270 (57%)
	MRI signal	114/360 (32%)
	Relationship to peritoneal reflection	152/360 (42%)
	T stage	340/360 (94%)
If ≥ T3	Distance through muscularis propria*	114/227 (50%)
MRF	MRF status	262/360 (73%)
	If threatened/involved, by what*	160/167 (96%)
	Minimum distance to the MRF*	96/151 (64%)
	Location closest to MRF	217/360 (60%)
If ≥ T4	Which organs involved*	75/83 (90%)
Nodes	Nodal status	348/360 (97%)
	If N+, location of involved nodes*	207/215 (96%)
	If N+, radial location of mesorectal nodal involvement*	125/206 (61%)
	If N+, superior location of node involvement*	69/204 (34%)
EMVI	EMVI status	224/360 (62%)
	EMVI radial and/or superior location*	71/115 (62%)
Metastases	Metastatic status*	107/244 (44%)
Overall predicted stage		329/360 (91%)

*Tumour descriptors with appropriate report exclusions allowed depending on the tumour features (e.g. the absence of a distance through the muscularis propria was considered acceptable for T1/T2 tumours or not stating which organs are involved by tumour with T1–3 staging, or radial location of wall involvement for annular tumours)

*MRF* mesorectal fascia, *EMVI* extra-mural venous invasion

## Impact of template reporting

Further analysis assessing the impact of template reporting showed statistically significant differences for most tumour descriptors after correction for multiple testing (Table 3). The only tumour descriptors with similar rates of inclusion in reports for free-text and template reports were tumour location, T-stage, descriptors of what threatened the MRF (i.e. tumour or lymph node), nodal status and location of involved nodes. The remaining tumour descriptors all demonstrated a statistically significant increase in report inclusion when a template was used. Most notably, this included relationship to the MRF that was only included in 184 of 279 (65.9%) of free-text reports compared to 78 of 81 (96.3%) of template reports (*p* < 0.01). Similarly, extra-mural venous invasion status was only included in 144 of 279 (51.6%) of the free-text reports compared to 80 of 81 (98.7%) of the template reports respectively (*p* < 0.01).

**Table 3:** Tumour descriptors and their inclusion on prose and template with statistical differences between the report styles included

		Total number of free-text reports including the variable/total number of free-text reports (%)	Total number of template reports including the variable/total number of template reports (%)	Fisher’s exact test *p* value	Critical *p* value
Tumour	Vertical location	248/279 (89%)	79/81 (98%)	0.0154	0.0100
	Length	233/279 (84%)	79/81 (98%)	0.0004	0.0050
	Distance from the anal verge	227/279 (81%)	78/81 (96%)	0.0004	0.0056
	Shape	181/279 (65%)	79/81 (98%)	0.0000	0.0033
	Radial location of wall involvement*	96/207 (46%)	60/63 (95%)	0.0000	0.0029
	MRI signal	36/279 (13%)	78/81 (96%)	0.0000	0.0023
	Relationship to peritoneal reflection	75/279 (26.9)	77/81 (95%)	0.0000	0.0024
	T stage	261/279 (94%)	79/81 (98%)	0.2684	0.0167
If ≥ T3	Distance through muscularis propria*	60/169 (36%)	54/58 (93%)	0.0000	0.0028
MRF	MRF status	184/279 (66%)	78/81 (96%)	0.0000	0.0036
	If threatened/involved, by what*	111/118 (94%)	49/49 (100%)	0.1069	0.0125
	Minimum distance to the MRF*	60/108 (56%)	36/43 (84%)	0.0013	0.0063
	Location closest to MRF	85/279 (31%)	58/81 (72%)	0.0000	0.0031
If ≥ T4	Which organs involved*	33/41 (81%)	42/42 (100%)	0.0024	0.0071
Nodes	Nodal status	268/279 (96%)	80/81 (99%)	0.3125	0.0250
	Location of involved nodes*	157/164 (96%)	50/51 (98%)	0.6835	0.0500
	Radial location of mesorectal nodal involvement*	81/156 (52%)	44/50 (88%)	0.0000	0.0045
	Superior location of node involvement*	25/156 (16%)	44/48 (92%)	0.0000	0.0025
EMVI	EMVI status	144/279 (52%)	80/81 (99%)	0.0000	0.0026
	EMVI radial and/or superior location*	37/79 (47%)	34/36 (94%)	0.0000	0.0042
Metastases	Metastatic status*	71/199 (36%)	36/45 (80%)	0.0000	0.0038
Overall predicted stage		249/279 (89%)	80/81 (99%)	0.0056	0.0083

*Tumour descriptors with appropriate report exclusions allowed depending on the tumour features (e.g. the absence of a distance through the muscularis propria was considered acceptable for T1/T2 tumours or not stating which organs are involved by tumour with T1–3 staging, or radial location of wall involvement for annular tumours)

*MRF* mesorectal fascia, *EMVI* extra-mural venous invasion

**Fig. 1:**
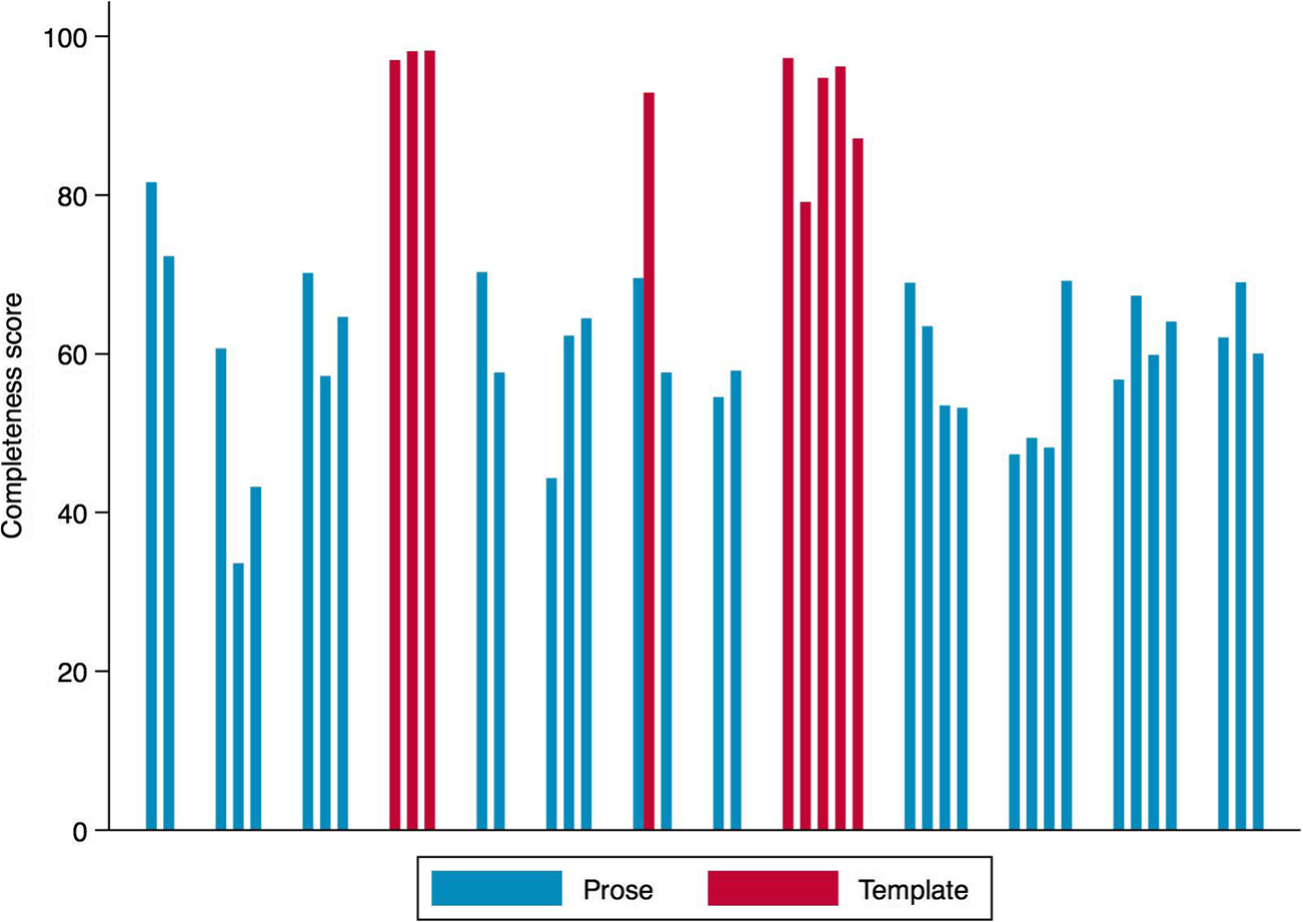
Bar chart of completeness scores (%) for each radiologist in the study. Red bars represent radiologists who have used template reports and blue bars radiologists that have used free-text (prose) reports. Each cluster of bars represents the radiologists within each department. Analysis of the completeness percentage score for template reports compared to free-text reports showed a median of 96% inclusion of all variables (IQR, 92–97%) compared to median 57% inclusion of all variables (IQR, 55–68%) respectively, *p* = 0.039

The median report scoring system demonstrated ‘completeness’ percentage across all reports was 65% inclusion of all variables (inter-quartile range, 57–72%). Subgroup analysis comparing template and free-text report groups demonstrated a significant improvement (*p* = 0.039) in the ‘completeness’ percentage score with template use, a median of 96% inclusion of all variables (IQR, 92–97%) compared to median 57% inclusion of all variables (IQR, 55–68%) respectively. Figure 1 shows the completeness percentage scores across all radiologists involved in the study.

## Discussion

This study has shown the current standard of primary staging rectal cancer MRI reports used in clinical practice consistently omit important information describing tumours. Here, in the largest study of its type, the use of template reporting has been shown to significantly improve the inclusion of key tumour descriptors when compared to free-text reporting in primary staging. A comparative study where the content of reports has been audited contains an assessment of only 128 reports and 11 tumour descriptors and did not assess the impact of template reports; by comparison, in our study, we have assessed 360 reports and 22 tumour descriptors including an assessment of the impact of template reports [21]. Here, it is shown the beneficial impact of template reports is similar to that observed in pathology reports for colorectal cancer [22–24]. Given that the majority of primary staging scans and reports are produced in regional hospitals rather than teaching hospitals, the same standards of reporting should be expected irrespective of the setting.

Alongside the significant improvement in the completeness percentage score for template reports compared to free-text reports, there was a reduction in spread of the interquartile range for the template report groups, with a reduction in the variability of template reports implying improved consistency for inclusion of key tumour descriptors. The inclusion of an increased number of tumour descriptors will inherently add important negative findings. Although the absence of tumour descriptors in free-text reports may imply a negative finding, the clear documentation of these may be helpful for clinicians and allow further studies assessing inter- and intra-radiologist agreement and correlation with pathological findings.

Several primary staging report templates have been developed with subtle differences in the tumour descriptors collected [14, 15, 25–27]. Irrespective of the template, we have assessed all of the key tumour descriptors included in each template recommended by the European and American abdominal imaging societies (ESGAR and SAR) [14, 15]. Although not acknowledged as key tumour descriptors, we have also assessed reports for descriptive information regarding the location of mesorectal fascia, metastatic lymph node and extra-mural venous involvement as these are reported locally to be helpful in surgical and radiotherapy planning. Template reporting significantly improves tumour description compared to free-text reporting styles, Table 3.

Despite a well-established prognostic link between tumour involvement of the MRF being associated with worse clinical outcomes due to the more frequent occurrence of a positive CRM, it is included in only 65.9% of free-text reports compared to 96.3% of template reports (*p* < 0.01). Subgroup analysis within this dataset, using only T3 or T4 tumours, shows inclusion of tumour involvement of the MRF is still only included in 81.1% of free-text reports compared to 100% of template reports (*p* < 0.01). The influence on prognosis of CRM involvement by the primary tumour compared to venous or lymphatic vessel invasion, or lymph nodes has not been fully evaluated other than two relatively small studies that demonstrated lymph node-CRM involvement had no impact on local recurrence, in distinction from other tumour components [28, 29]. However, these tumour descriptors are recorded in the SAR guideline template [15]. We found there was no difference in report inclusion of what aspect of the primary tumour threatened the MRF in each report style. As a potentially important variable to assess in relation to the prognosis, its inclusion in template reports could help to better understand its value.

Furthermore, there are subtle differences between the method of sub-categorisation for T3 tumours within the ESGAR and SAR templates [14, 15]. The ESGAR template dichotomises T3 tumours into two groups (either ≤ 5 mm or ≥ 5 mm extra-mural growth beyond the muscularis propria) [14]. By comparison, the SAR template uses four T3 sub-categories (a, < 1 mm; b, < 5 mm; c, 5–15 mm or d, ≥ 15 mm) for tumour penetration beyond the muscularis propria. Each system stratifies patients, which influences the neo-adjuvant therapies offered based on prior studies demonstrating the prognostic significance of the depth extra-mural of tumour extension on locoregional recurrence [28, 30, 31]. There is conflicting evidence as to the precise depth that is significant for an increased risk of locoregional recurrence, either 5 mm or 10 mm beyond the muscularis propria [28, 30, 31]. However, irrespective of the sub-categorisation method used, this study demonstrates the use of template reports significantly increases documentation of depth of invasion beyond the muscularis propria (93.1% vs 35.5% inclusion of T3 subcategories/depth in template vs free-text reports, *p* < 0.01).

Accurately determining tumour involvement in lymph nodes based on size and morphological appearance can be difficult in rectal cancer MRI, but the description of involved/potentially involved lymph nodes is undeniably important [32]. In our population the use of a template did not improve reporting on nodal status (96.1% inclusion in free-text reports compared to 98.8% inclusion in template reports; corrected *p* < 0.3125) or descriptions of intra- or extra-mesorectal node location (95.7% inclusion in free-text reports compared to 98.0% inclusion in template reports; corrected *p* < 0.68). One limitation of our study is that neither the free-text nor template reports assessed if established criteria were applied to determine if nodes were involved with tumour or not. Template reports allow more categorisation of the rationale for determining nodal status; for example based on size and/or other morphological features as in the ESGAR and SAR consensus templates [14, 15]. Further assessment of the features and pathological correlation may improve radiologist and clinician confidence in determining which nodes are involved with malignant disease.

The retrospective design of this study assesses the current reporting standards of primary staging for rectal cancer in routine clinical practice in 2017 provided by subspecialised GI radiologists. It highlights potential areas for quality improvement and standardisation through the use of template reports despite subspecialised GI training for radiologists reporting large volumes of rectal cancer staging MRI. Unlike the study by Siddiqui et al, the existing use of the template reports in two centres (by nine radiologists) eliminates the potential bias arising from the introduction and associated training with template reports when assessing their impact [26, 33]. However, to maximise the benefits of template reports in our population, their introduction to free-text reporting centres should occur in conjunction with appropriate training that would reiterate the importance of key tumour descriptors. Furthermore, here we have demonstrated that template reports include more tumour descriptors than free-text reports. Further work is required to demonstrate that report accuracy and inter-radiologist agreement is maintained or even improves with standardised descriptive terms found in template reports.

A limitation of this study is the relatively small number of template reports used within two trusts, compared to free-text reports. Although this might increase the likelihood of a type 1 statistical error in assessing the impact of template reports, we have primarily assessed the current standard of primary staging reports irrespective of template use. Additionally, because our findings are replicated across multiple tumour descriptors and show strong statistical significance after correction, multiple type 1 errors are unlikely.

Primary staging rectal cancer MRI reports in routine clinical practice do not meet published standards with multiple key tumour descriptors omitted from reports. A standardised report template results in a significant increase in the inclusion of key tumour descriptors for subspecialised GI radiologists. This study provides further support for the routine use of template reports to improve reporting standards and outcomes in rectal cancer.

## Abbreviations

BSGAR: British Society of Gastrointestinal and Abdominal Radiology
CRC-MDTs: Colorectal cancer multi-disciplinary teams
CRM: Circumferential resection margin
CRT: Chemoradiotherapy
EMVI: Extra-mural venous invasion
ESGAR: European Society of Gastrointestinal Abdominal Radiology
GI: Gastrointestinal
MRF: Mesorectal fascia
MRI: Magnetic resonance imaging
SAR: Society of Abdominal Radiology
UK: United Kingdom
